# High-throughput and automated diagnosis of antimicrobial resistance using a cost-effective cellphone-based micro-plate reader

**DOI:** 10.1038/srep39203

**Published:** 2016-12-15

**Authors:** Steve Feng, Derek Tseng, Dino Di Carlo, Omai B. Garner, Aydogan Ozcan

**Affiliations:** 1 Electrical Engineering Department, University of California, Los Angeles, California 90095, United States; 2 Bioengineering Department, University of California, Los Angeles, California 90095, United States; 3 California NanoSystems Institute, University of California, Los Angeles, California 90095, United States; 4 Jonsson Comprehensive Cancer Center, University of California, Los Angeles, California 90095, United States; 5 Department of Pathology and Laboratory Medicine, David Geffen School of Medicine, University of California, Los Angeles, California 90095, United States; 6 Department of Surgery, David Geffen School of Medicine, University of California, Los Angeles, California 90095 United States

## Abstract

Routine antimicrobial susceptibility testing (AST) can prevent deaths due to bacteria and reduce the spread of multi-drug-resistance, but cannot be regularly performed in resource-limited-settings due to technological challenges, high-costs, and lack of trained professionals. We demonstrate an automated and cost-effective cellphone-based 96-well microtiter-plate (MTP) reader, capable of performing AST without the need for trained diagnosticians. Our system includes a 3D-printed smartphone attachment that holds and illuminates the MTP using a light-emitting-diode array. An inexpensive optical fiber-array enables the capture of the transmitted light of each well through the smartphone camera. A custom-designed application sends the captured image to a server to automatically determine well-turbidity, with results returned to the smartphone in ~1 minute. We tested this mobile-reader using MTPs prepared with 17 antibiotics targeting Gram-negative bacteria on clinical isolates of *Klebsiella pneumoniae,* containing highly-resistant antimicrobial profiles. Using 78 patient isolate test-plates, we demonstrated that our mobile-reader meets the FDA-defined AST criteria, with a well-turbidity detection accuracy of 98.21%, minimum-inhibitory-concentration accuracy of 95.12%, and a drug-susceptibility interpretation accuracy of 99.23%, with no very major errors. This mobile-reader could eliminate the need for trained diagnosticians to perform AST, reduce the cost-barrier for routine testing, and assist in spatio-temporal tracking of bacterial resistance.

The increasing prevalence of antimicrobial resistance represents a severe threat to global health^1^,^2^ and is becoming more common to bacterial pathogens in high mortality diseases including pneumonia^3^, diarrheal disease^4^, and sepsis^5^. Part of the global challenge in combating these organisms is that routine antimicrobial susceptibility testing (AST) is not often performed due to technological challenges, high costs, and lack of professional training, which greatly contributes to high mortality and the global spread of multi-drug resistant organisms^6^,^7^,^8^,^9^,^10^. The goals of antimicrobial susceptibility testing include the detection of possible drug resistance and assurance of susceptibility to drugs of choice for each particular infection.

The gold standard for antimicrobial susceptibility testing is the broth microdilution method^11^. This procedure involves preparing two-fold dilutions of antibiotics in a liquid growth medium that is dispensed in a 96-well microtiter plate (MTP), with plates typically prepared for standard bacterial groups (e.g., Gram-negative or Gram-positive). The antibiotic containing wells are inoculated with a standardized bacterial suspension with bacteria isolated from a patient. Following overnight incubation, the tubes are examined by trained experts for visible growth as evidenced by turbidity (see Fig. 1a,c). The lowest concentration of antibiotic that prevented growth represents the minimum inhibitory concentration (MIC). This is a *quantitative* result that allows tracking of resistance. The MIC value must be interpreted using a table of values that relate to the proven clinical efficacy of each antibiotic for various bacterial species. An interpretive criterion (e.g., *susceptible* or *resistant*) is assigned to each bacteria/drug combination in order to guide the physician in treatment decisions. These interpretive criteria have been established by both the U.S. Food and Drug Administration (FDA) and the Clinical Laboratory Standards Institute (CLSI) using data from animal studies, microbiological studies, and clinical efficacy data. A susceptible result indicates that the patient’s organism should respond to therapy, while a resistant organism will not be inhibited by the concentrations of antibiotic achieved with normal dosages used for that drug.

An important part of the global challenge for this gold standard testing is that a high level of clinical microbiology expertise and tedious examination of the well plate is required to read the turbidity from the MTP and to establish the interpretive criteria necessary for treatment. An additional global challenge is that the data collected in clinical microbiology laboratories are not easily available for epidemiological studies, and are not available at all in regions of the world where antimicrobial susceptibility testing is not regularly performed.

Over the past several years, smartphones and other portable consumer electronics devices have demonstrated their capability for serving as a multiplexed platform for building biomedical imaging, sensing and diagnostic systems^12^,^13^,^14^,^15^,^16^,^17^,^18^,^19^,^20^,^21^,^22^,^23^,^24^,^25^,^26^,^27^,^28^,^29^,^30^,^31^,^32^,^33^,^34^,^35^,^36^,^37^,^38^,^39^,^40^,^41^,^42^. These portable measurement tools typically utilize opto-mechanical attachments used in conjunction with the built-in capabilities of the mobile device (e.g., camera, microphone, GPS) to provide cost-effective, rapid, portable, and highly distributable imaging and sensing systems. As an example, we previously developed a cellphone-based 96-well microplate reader for analyzing enzyme-linked immunosorbent assays (ELISA) and demonstrated its capability for reading and quantification of various FDA-approved colorimetric ELISA tests including e.g., mumps IgG, measles IgG, and herpes simplex virus IgG^29^. Using the same opto-mechanical hardware attachment to a mobile phone and a new custom-designed smart application and computational approach, here we report a new cellphone-based antimicrobial susceptibility testing system that can serve as a cost-effective, hand-held, and automated turbidity reader for rapid quantification and analysis of micro-well susceptibility results (see Fig. 2). This AST system is composed of a smartphone (Nokia Lumia 1020) that is paired with a 3D printed portable opto-mechanical attachment and a data processing server (e.g., a laptop or a PC) in communication with a Windows Phone based mobile application that serves as an interactive graphical user interface (GUI). After inserting an antimicrobial susceptibility test plate into the attachment, the phone’s camera application is used to capture the transmitted light from each well in the MTP at multiple exposures (see Fig. 1b,d), and then our app uploads these images to a local or remote server to automatically quantify well turbidity. By selecting the drug target type of the treated plate (e.g., Gram-negative or Gram-positive) and the microbe of interest, the server then determines the drug-specific MIC and corresponding interpretive criteria and returns the results to the user through the same mobile application within 1 minute.

We demonstrated this mobile system’s ability to automatically determine drug-specific MIC and corresponding drug resistance through a comprehensive clinical evaluation performed at the UCLA Clinical Microbiology Laboratory using plates containing 17 different antibiotics targeted for Gram-negative bacteria and tested on patient isolates of *Klebsiella pneumoniae*. This species of bacteria can exhibit highly resistant antimicrobial profiles and contain members of the Carbapenem resistant *Enterobacteriaceae* (CRE), with a very high mortality rate in multiple disease states including sepsis and pneumonia^43^,^44^,^45^,^46^,^47^,^48^,^49^,^50^. CRE have complicated antimicrobial resistance profiles and represent a significant challenge to global health. Our mobile AST reader’s performance exceeds the FDA defined criteria for susceptibility testing^51^ with an MIC agreement of >95% with no very major errors (i.e., resistant microbes misdiagnosed as susceptible), 0.16% major errors (i.e., susceptible microbes misdiagnosed as resistant), and 0.65% minor errors (i.e., indeterminate/susceptible dose dependent-related misdiagnoses).

## Methods

### System Design

**Figure 1:**
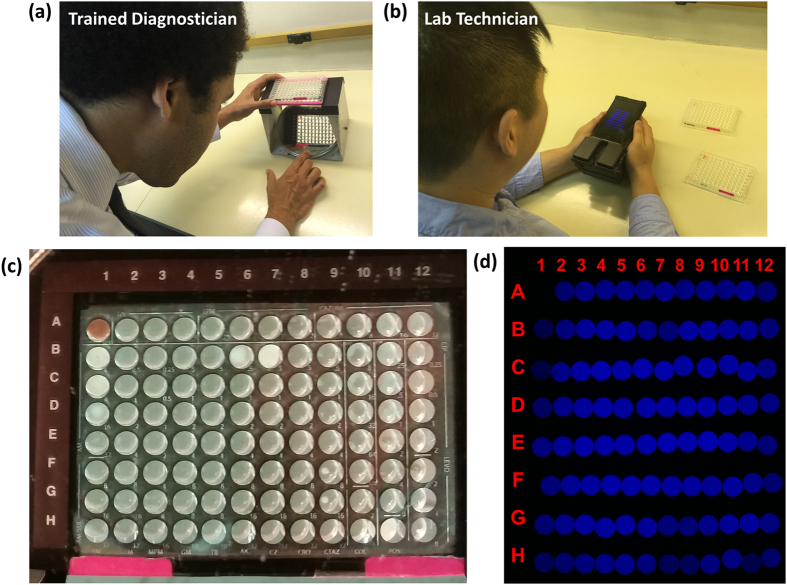
Comparison of existing method of diagnosis and our method. (**a**) Current diagnosis method consists of diagnosticians manually inspecting plates under a light source to identify well turbidity as a sign of microbial growth. In this case, a mirror is used to provide ease of viewing the plate. (**b**) When using our system, a lab technician untrained in plate diagnosis will only need to take three images of each plate and upload the captured images using our application to a local or remote server for automated processing to obtain equivalent diagnostic results. (**c**) A close-up of the mirrored image. Here wells B1, C1, D1, B6, B7, and H11 contain turbidity. (**d**) The same plate as imaged using our system. The presence of turbidity in each well is automatically determined based off the measured transmittance of light through the well.

**Figure 2:**
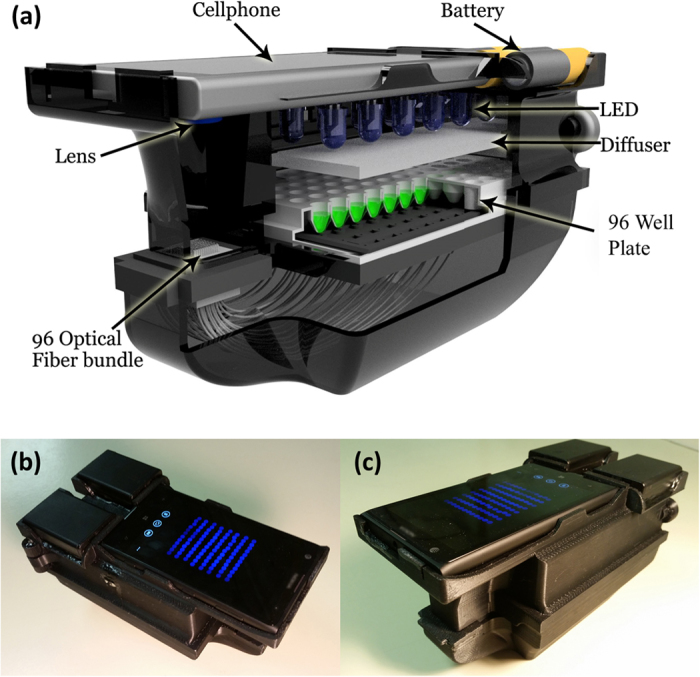
Portable 96-well microtiter plate turbidity reader for antimicrobial susceptibility testing. (**a**) Our device consists of a custom-design hardware attachment for the Nokia Lumia 1020 smartphone, using an LED array through a diffuser to illuminate 96-well plates, which are optically transmitted through an optical fibre bundle to a small region of interest, imaged through the smartphone’s built-in camera module with the addition of a focusing lens. (**b**) Top view of our device. (**c**) Side view of our device.

Our AST system (Fig. 2) is comprised of a Windows Phone-based smartphone used to capture images of the MTP, held within a 3D-printed attachment including optical components for illuminating the MTP, and a local or remote server for rapidly processing and interpreting the plate images uploaded through a custom-designed application running on the same smartphone. While our current prototype is based on a Nokia Lumia phone, this platform can also be used with iOS and Android based smartphones with minor modifications to its design. Figure 2a provides a cross-section of our 3D printed device, with the cellphone placed at the top, next to six AAA batteries, which power an array of 24 uniformly distributed light-emitting-diodes (LEDs, at 464 nm) through a low-noise, low-dropout linear current regulator to prevent power fluctuations and maintain constant illumination of the AST plate^29^. To maximize the spatial uniformity of the illumination, each LED is centered between 4 wells and placed above two layers of optical diffusers to homogenize the illumination. When testing a new plate, the MTP is inserted into a fitted space below the LED array, with an inexpensive plastic-based fiber-optic bundle coupling the blue LED light that is transmitted through each well to an external lens (focal length 45 mm) placed in front of the cellphone camera. The smartphone’s camera application is used to obtain raw 10-bit Digital Negative (DNG) format images of the fiber bundle as seen in Fig. 1d. This unique instrument design involving a fiber-optic array that is mapped to individual wells of a plate provides a significantly more compact and light-weight AST platform without suffering from optical aberrations since the 96-well plate is transformed into an imaging area at the end of the fiber bundle that is more than two orders of magnitude smaller compared to the actual area of the well plate. Bending induced losses in each fiber optic cable can create well-to-well signal variations, all of which have been calibrated out after the assembly of the fiber-optic array by using blank plates. The overall dimensions of this hand-held AST reader are ~195 × 98 × 100 mm (see Fig. 2) and it weighs ~0.62 kg. All the components of our opto-mechanical attachment to the smartphone would cost ~100 USD in total, even at very low production volumes. An automated AST platform, on the other hand, typically costs significantly more (e.g., ~$30,000) and would be much larger and heavier compared to our hand-held platform, although it would also perform other AST related functions including e.g., the Kirby-Bauer Disk diffusion interpretation, among other automated tasks that we do not perform in our mobile platform.

### Image Analysis for AST

**Figure 3:**
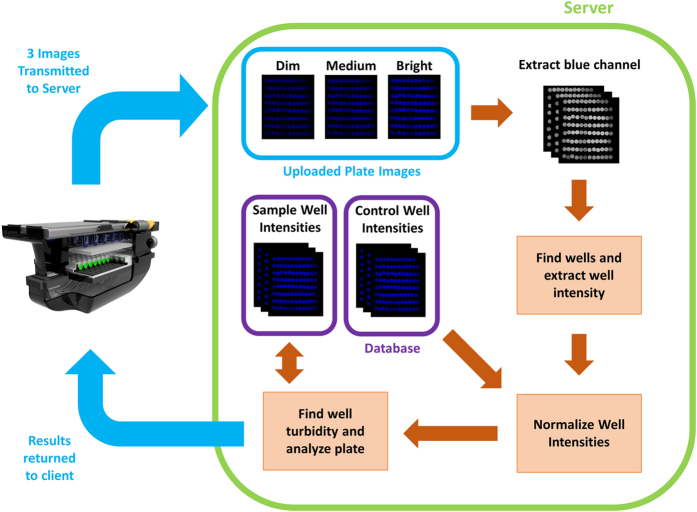
Overview of client-server communication and image analysis. Using our custom-developed mobile application, three images of the sample plate taken at different exposure times are uploaded to our local or remote server. Our automated algorithm finds the wells using the blue channel of the brightest exposure image and extracts the average well intensity. The well intensities for the three exposures are then combined to maximize the dynamic range of our measured intensities and normalized respective to the maximum transmittance as obtained from blank plate control wells. These well intensities are then thresholded based off a database of uploaded plates and processed to determine each drug’s minimum inhibitory concentration and susceptibility. These results are stored on the server along with the uploaded plate images, and also returned to the client application to be shown to the user.

**Figure 4:**
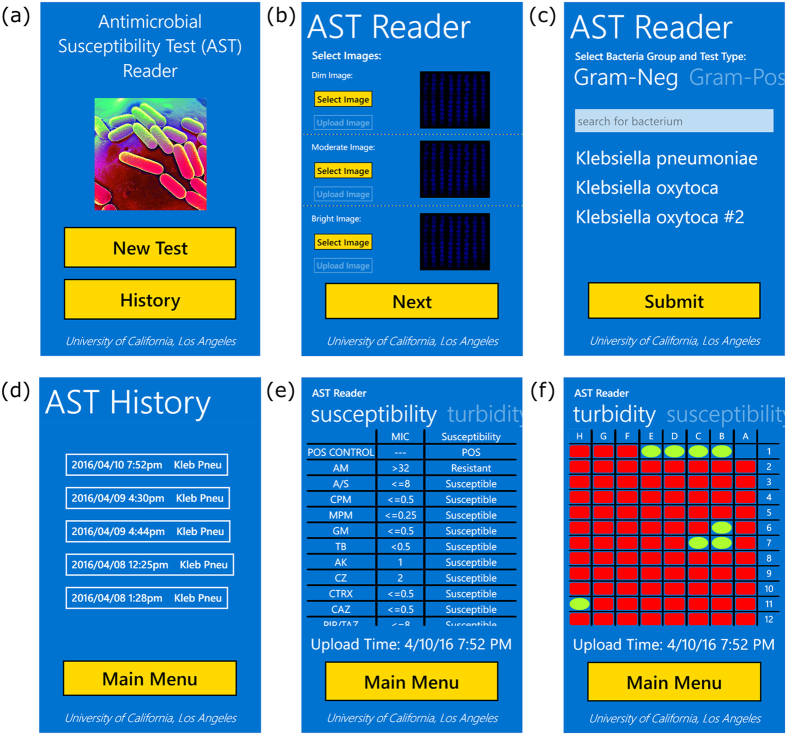
Screenshots of our custom Windows Phone application for uploading images of well plates and displaying processed results. (**a**) From the main menu, users can submit a new test or review previously submitted tests. (**b**) After starting a new test, users select and upload three DNG format images of the 96-well plate captured at 3 different exposures. (**c**) Users next select the type of plate to be processed and choose the microbe to-be-tested. A search bar facilitates the selection process, after which users select the specific microbe. Clicking the “submit” button sends the plate type, microbe, and images to a local or remote server for processing. (**d**) Processed results can be reviewed through a history listing based off the time of upload and plate type. (**e,f**) Plate analysis returns the minimum inhibitory concentration and the corresponding drug susceptibility diagnosis for the specified plate type in a scrollable table. The turbidity detection for each well can also be viewed in a colored table, with red rectangles indicating no turbidity and green-yellow ovals indicating wells with turbidity.

Figure 3 illustrates the communication between our smartphone application and the data processing server, which is also used for spatio-temporal tagging and storage of our results. After inserting a new AST plate into the attachment, images are captured using the mobile phone’s camera application at three different exposure times (dim: 1/1600 sec, moderate: 1/1250 sec, and bright: 1/800 sec) and saved as 10-bit DNG images. These 10-bit DNG images represent the best possible image quality obtainable on this mobile platform, with the other alternative being compressed JPEG format with 8-bit images. From the main menu (see Fig. 4a), the user starts a new test analysis and first selects the three images previously captured to be used for processing (see Fig. 4b). Next, the user selects whether the plate type is Gram-negative or Gram-positive and then uses the search box to find the bacterium to-be-tested, such that the server knows which wells correspond to which drugs and how to analyse the MIC and perform automated drug susceptibility interpretation, respectively. By clicking “submit”, the images, plate type, and bacterium to-be-tested are sent to a local or remote server for processing.

On receiving a new request, the server (implemented in Python using the Twisted framework) adds the request to the job queue and saves the images and job details to local storage. Each DNG image is converted into 16-bit tagged image file format (TIFF) and the pixels corresponding to the blue channel are extracted from the raw Bayer image. Subsequent processing of the blue channel images is performed using MATLAB. The server also has pre-recorded information of the mapping between the fiber-optic cables of our AST device and the individual wells of a 96-well plate. This 2D mapping does not change from image to image, and only needs to be determined once for a given device design. To find this mapping function, a bright-field image (i.e., a control image) of a plate is taken with deionized (DI) water with no turbidity in the wells and the centre of each fiber-optic cable is digitally calculated. To map successive images of the AST plates to this control image, corner wells are found using a threshold-based approach followed by morphological operations to separate adjacent wells. The corner wells are then used to remove scaling and alignment issues that might be caused by the camera’s auto-focus feature and potential misalignments between the camera and fiber-optic array, where the known physical distances among the fiber-optic cables are exploited to find the centre of each well. A circular mask with a radius of 25 pixels is then applied to reduce the interference from nearby fiber-optic cables before extracting the average intensity of each well’s signal.

After extracting each well’s pixel intensity, the average intensities per well for the dim, moderate, and bright images are each normalized with respect to the average intensities of control images at each exposure time to scale the intensities from 0 to 1, with 1 representing complete transmittance of light through the well relative to the DI control. To maximize our dynamic range, the normalized transmittance values for each well from the dim, moderate, and bright images are first scaled respective to their integration times and then averaged to obtain a single higher dynamic range value for each well.

To determine whether a well contains turbidity or not, we use a threshold-based approach to determine the cut-off transmittance value for each well. To choose an accurate threshold value for each well, we extract the normalized transmittance values of wells with no turbidity from a training set of patient plates and blank reference plates (i.e., plates with drugs but no microbes). As the turbidity increases, the light transmittance through the well decreases. Thus, from this set of normalized transmittance values from wells with no turbidity, for each well we decided to use a threshold at two times the standard deviation below the mean to determine whether a given well is statically likely to have microbial growth represented by turbidity. For a given plate type (i.e., Gram-negative or Gram-positive), the MIC for each drug is determined by finding the *first well* in each drug-specific set of wells that contains turbidity. Depending on the selected microbe of interest, drug susceptibility interpretations are automatically made based on the MIC for each drug (see Supplementary Table 1 for an example chart for *Klebsiella pneumoniae*).

After the turbidity decisions of the wells, the MIC determination, and the drug susceptibility interpretations are all automatically made, the results are stored in a database on the server, and are also sent back to the originating smartphone within 1 minute. On the smartphone application, the user can review the results via the history page (see Fig. 4d), which lists all the uploaded tests and the microbes tested for. After clicking on a test, the user can view the susceptibility results in a scrollable table (see Fig. 4e), with the drug or drug combination in the first column, the MIC in the second column, and the susceptibility interpretation in the third column. The user can also swipe the page to see the detection of well turbidity in a color-coded table format (see Fig. 4f), with red coloured rectangles indicating no turbidity and green-yellow coloured ovals indicating the wells that contain turbidity.

### Design of Clinical Testing

We tested our platform using *Klebsiella pneumonia* isolates from patient samples collected by the UCLA hospital system and prepared and tested at the UCLA Clinical Microbiology Laboratory. Antimicrobial agents are tested using two-fold serial dilutions and the concentration range varies with the drug, the organism tested, and the site of the infection. For the microdilution method, the antimicrobial dilutions are in 0.1 mL volumes that are contained in wells of a 96 well microdilution tray. The drug panels are then stored frozen until they are inoculated. Briefly, a suspension of the tested organism is prepared in sterile saline to a 0.5 McFarland standard using isolated colonies. 1.5 mL of the suspension is transferred to an inoculating tray containing 40 mL of sterile distilled water. The inoculating tray has prongs that allow for transfer of bacteria into each well of the 96 well drug plate. The plate is then incubated for 24 hours at 37 °C. The panels are quality controlled with the appropriate ATCC (American Type Culture Collection) organisms. The bacterial pathogen identification was performed after the culture of the organism by MALDI-TOF (matrix assisted laser desorption/ionization - time of flight) identification method. For each plate, an expert diagnostician inspected the plate and recorded the presence or absence of turbidity in each well, which was used as our gold standard. Each plate was then imaged using our smartphone-based platform. All experiments were conducted at the UCLA Clinical Microbiology Laboratory by a medical personnel trained on how to use the mobile platform. This study was found to be exempt from IRB (Institutional Review Board) review by the UCLA Office of the Human Research Protection Program.

### Calculation of MIC and Drug Susceptibility Interpretation

The MIC and drug susceptibility for *Klebsiella pneumoniae* were determined using a chart provided by the UCLA Clinical Microbiology Laboratory (see Supplementary Table 1). For each drug, the MIC is determined by finding the first well with turbidity for increasing drug concentration and the susceptibility determined using a look-up table.

## Results

We validated the capability of our cellphone-based AST system to perform highly accurate MIC determination and drug susceptibility interpretation, greatly exceeding the FDA-defined criteria for susceptibility testing, with clinical isolates of the Gram-negative bacterium *Klebsiella pneumoniae*. Table 1 shows the mean and standard deviation for well turbidity detection accuracy, well turbidity detection sensitivity, well turbidity detection specificity, MIC determination accuracy, and drug susceptibility interpretation accuracy of our AST reader when using only the best performing single exposure image (i.e., bright exposure) and when combining the dim, moderate, and bright exposure images to digitally increase the dynamic range. In these trials, 39 randomly chosen patient isolate plates and 21 blank plates without microbial content were used to determine an optimal threshold for well turbidity detection, followed by a blind-test on the remaining 39 patient isolate plates, none of which were used in our training. Blank plates were included in the training set since some wells always exhibit bacterial growth due to high antimicrobial resistance. This training and blind-testing process was performed 50 times with random sampling of patient plates to generate the standard deviations. As can be seen from this table, combining multiple image exposures significantly increases the overall accuracy of AST using our system and reduces variability for well turbidity detection, with significant improvements for MIC determination and drug susceptibility interpretation. Based on these results, we achieved an average well turbidity detection accuracy of 98.21%, a minimum inhibitory concentration accuracy of 95.12%, and a drug susceptibility interpretation accuracy of 99.23%, with no very major errors (i.e., resistant misdiagnosed as susceptible), 0.16% major errors (i.e., susceptible misdiagnosed as resistant), and 0.65% minor errors (i.e., indeterminate/susceptible dose dependent-related misdiagnoses). To provide a reference frame for these numbers, our total ground truth dataset across 78 patient plates contains 960 susceptible decisions, 288 resistant decisions, 70 indeterminate decisions, and 8 susceptible dose dependent decisions.

To better explore potential drug susceptibility misdiagnoses using our system, Table 2 shows the specific results for one training/test set of the multiple exposure results used in the statistical average reported in Table 1. Due to the design of the Gram-negative MTP used by UCLA Clinical Microbiology Laboratory (see Supplementary Table 1), only 95 wells are used per plate, with 1 well used as a positive control, 1 well used as a positive dye, and the remaining 93 wells used for drug testing, which provides us a total of 3705 turbidity-assessable wells across 39 patient isolate test plates used in our blind testing. These wells are used to test 17 drugs and drug combinations per MTP (see Supplementary Table 2 for a list of all the drugs used in our experiments) for a total of 663 MIC determinations and drug susceptibility interpretations. We note that in this particular example reported in Table 2, the automated turbidity detection and corresponding MIC and drug susceptibility accuracy (see Table 2) performed similarly to the average system performance exhibited in Table 1. Additionally, this example reported in Table 2 reveals that there are no very major errors (i.e., no resistant bacteria misdiagnosed as susceptible) and only 1 major error (i.e., ~0.2% susceptible bacteria misdiagnosed as resistant) and 2 minor errors (i.e., ~0.3% indeterminate/susceptible dose dependent-related misdiagnoses) out of a total of 663 MIC and drug susceptibility interpretations across 39 patient test plates (see Table 2), with no error occurring twice on the same plate or for the same drug, exceeding the FDA criteria for clinical susceptibility testing.

## Discussion

We demonstrated a cost-effective portable system composed of a mobile phone and a 3D printed opto-mechanical attachment that can replace an expert diagnostician with a lab technician trained in the usage of this device for interpreting 96-well microtiter plates for antimicrobial susceptibility testing. This mobile platform achieved 95.12% MIC determination accuracy and 99.23% drug susceptibility interpretation accuracy for *Klebsiella pneumonia* susceptibility testing, exceeding the FDA criteria for performing AST analysis. Since well turbidity presents similar optical characteristics, adding the ability to test other plate types and microbes can be as simple as updating the server logic with the drug series information and drug-microbe susceptibility interpretation as per Supplementary Table 1, allowing this platform to easily scale to test other bacteria.

One limitation of this technology remains the need for preparation of the 96-well microtiter plate, which typically requires a large machine to deposit specific drug concentrations in each well and fill each well with the microbes to-be-tested, extracted from a patient. Regardless, this technology is especially useful in resource-limited settings given its ability to remove the need for a trained diagnostician, enabling local technicians to easily be able to conduct high-throughput antimicrobial susceptibility testing. In fact, clinical microbiology is rapidly progressing toward automation. Multiple platforms are now available for automated organism identification including smart incubators and MALDI-TOF based proteomic identification. Our results and the demonstrated platform fit very well into future clinical microbiology diagnostic labs, where the gold standard for AST testing and broth microdilution can be automated for turbidity reading, MIC interpretation, and appropriate antibiotic prescription. Furthermore, paired with the smartphone’s wireless connectivity and inherent digital record-saving, this platform can enable widespread and easy collection of drug resistance profiles for spatio-temporal tracking, which could be especially useful for isolating and eliminating drug resistant strains of harmful microbials. An additional advantage of this technology is the possibility of examining turbidity or bacterial growth in the presence of a drug at an earlier time point than is currently read (24 hours). Optical analysis by the digital reader may potentially reveal early turbidity and allow for a more rapid turn-around time of the AST results to the physician.

## Additional Information

**How to cite this article**: Feng, S. *et al*. High-throughput and automated diagnosis of antimicrobial resistance using a cost-effective cellphone-based micro-plate reader. *Sci. Rep.* **6**, 39203; doi: 10.1038/srep39203 (2016).

**Publisher's note:** Springer Nature remains neutral with regard to jurisdictional claims in published maps and institutional affiliations.

## Supplementary Material

Supplementary Information

## Figures and Tables

**Table 1:** Random sub-sampling validation results averaged over 50 trials, for well turbidity analysis and subsequent determination of MIC and microbial drug susceptibility with very major (i.e., resistant microbes diagnosed as susceptible), major (i.e., susceptible microbes diagnosed as resistant), and minor (i.e., indeterminate/susceptible dose dependent-related misdiagnoses) error percentages for our algorithm using (a) a single exposure image and (b) a combination of three exposures.

Images used	Well Accuracy	Well Sensitivity	Well Specificity	MIC Accuracy	Drug Susceptibility Accuracy	Very Major Error Percentage	Major Error Percentage	Minor Error Percentage
(a) Single exposure (1/800)	96.94 ± 1.30%	98.83 ± 0.70%	96.24 ± 1.92%	94.89 ± 1.14%	98.55 ± 0.58%	0.43 ± 0.68%	0.17 ± 0.13%	1.23 ± 0.54%
(b) Combination of three exposures (1/1600, 1/1250, 1/800)	98.21 ± 0.29%	98.56 ± 0.37%	98.08 ± 0.37%	95.12 ± 0.87%	99.23 ± 0.23%	0 ± 0%	0.16 ± 0.18%	0.65 ± 0.20%

78 patient isolate testing plates from the UCLA Hospital Microbiology Lab and 21 blank plates without microbial content were randomly separated 50 times into training sets of 21 blank plates and 39 patient plates and blind-test sets of 39 patient plates. The threshold is determined using the training set and run on the corresponding test set, with mean and standard deviation across the 50 trials shown in the table. Combining multiple image exposures significantly increases the overall accuracy and reduces the variability for well turbidity detection, with corresponding improvements for MIC determination and drug susceptibility interpretation as well as significant reductions for very major, major, and minor errors.

**Table 2:** Trial results from using a training set of 21 blank plates and randomly chosen set of 39 patient isolate sample plates and blind-test set of remaining 39 samples from a total of 78 *Klebsiella pneumoniae* patient isolate plates.

(A) System Performance				
Well Accuracy			98.25%	
Well Sensitivity			98.27%	
Well Specificity			98.23%	
MIC Accuracy			95.32%	
Drug Susceptibility Accuracy			99.55%	
Very Major Error Percentage			0%	
Major Error Percentage			0.21%	
Minor Error Percentage			0.30%	
**(B) Drug Susceptibility Misdiagnoses**				
**Plate ID**	**Drug**	**Automated System Decision**	**Diagnostician Decision**	**Error Type**
1	AM	Resistant	Indeterminate	Minor
5	A/S	Resistant	Indeterminate	Minor
8	MPM	Resistant	Susceptible	Major

There are 95 applicable wells per plate, with 1 well used as a positive control, 1 well as a positive dye, and the remaining 93 wells for drug testing, yielding a total of 39 × 95 = 3705 applicable wells assessed for turbidity. Each plate tests 17 different drugs and drug combinations for a total of 663 MIC and drug susceptibility decisions. As highlighted in our System Performance summary (A), well turbidity, MIC and drug susceptibility decisions are all comparable to the average performance of our system reported in Table 1. The three specific drug susceptibility misdiagnoses reported in (B) (i.e., plates 1, 5 and 8) are all low-risk misdiagnoses, with 1 major error and 2 minor errors but *no very major errors*. None of these are for the same drug or plate.
