# Supplementary material for: High-throughput and automated diagnosis of antimicrobial resistance using a cost-effective cellphone-based micro-plate reader
Source: Sci Rep. 2016 Dec 15;6:39203. doi: 10.1038/srep39203 (PMC5156953; doi:10.1038/srep39203)
Supplement: Supplementary Information [file srep39203-s1.pdf]

Supplementary Information for

High-throughput and automated diagnosis of antimicrobial resistance using a cost-effective cellphone-based micro-plate reader

Steve Feng,[1,2] Derek Tseng,[1,2] Dino Di Carlo,[2,3,4] Omai Garner,[5] and Aydogan Ozcan[1,2,3,6*]

[1] Electrical Engineering Department, University of California, Los Angeles, California 90095, United States

[2] Bioengineering Department, University of California, Los Angeles, California 90095, United States

[3] California NanoSystems Institute, University of California, Los Angeles, California 90095, United States

[4] Jonsson Comprehensive Cancer Center, University of California, Los Angeles, California 90095, United States

[5] Department of Pathology and Laboratory Medicine, David Geffen School of Medicine, University of California, Los Angeles, California 90095, United States

[6] Department of Surgery, David Geffen School of Medicine, University of California, Los Angeles, California 90095 United States.

* E-mail: ozcan@ucla.edu ; Fax: +1 310 206-4833; Tel: +1 310 825-0915

	1	2	3	4	5	6	7	8	9	10	11	12
A	---	TMP/SMX 1/20	TMP/SMX 2/40	TMP/SMX 4/80	ETP 0.25	ETP 0.5	ETP 1	ETP 2	CAZ/AVI 4	CAZ/AVI 8	CAZ/AVI 16	CAZ/AVI 32
B	AM 4	CPM 0.5	MPM 0.25	GM 0.5	TB 0.5	AK 0.5	CZ 0.5	CTRX 0.5	CAZ 0.5	PIP/TAZ 8	IPM 0.25	CIP 0.25
C	AM 8	CPM 1	MPM 0.5	GM 1	TB 1	AK 1	CZ 1	CTRX 1	CAZ 1	PIP/TAZ 16	IPM 0.5	CIP 0.5
D	AM 16	CPM 2	MPM 1	GM 2	TB 2	AK 2	CZ 2	CTRX 2	CAZ 2	PIP/TAZ 32	IPM 1	CIP 1
E	AM 32	CPM 4	MPM 2	GM 4	TB 4	AK 4	CZ 4	CTRX 4	CAZ 4	PIP/TAZ 64	IPM 2	CIP 2
F	A/S 8	CPM 8	MPM 4	GM 6	TB 6	AK 8	CZ 8	CTRX 8	CAZ 8	PIP/TAZ 128	IPM 4	LVX 2
G	A/S 16	CPM 16	MPM 8	GM 8	TB 8	AK 16	CZ 16	CTRX 16	CAZ 16	PIP/TAZ 256	IPM 8	LVX 4
H	A/S 32	CPM 32	MPM 16	GM 10	TB 10	AK 32	CZ 32	CTRX 32	CAZ 32	POS CNTRL	DYE	LVX 8

= susceptible   = susceptible dose dependent (SDD)   = indeterminate   = resistant

Supplementary Table S1. Standard drug concentrations populated in each well for a Gram-negative 96-well microtiter plate at the UCLA Medical Center and color-coded with interpretive criteria readings for *Klebsiella pneumoniae*. Green is susceptible, yellow is intermediate, orange is susceptible dose dependent, and red is resistant. The minimum inhibitory concentration (MIC) of each drug for the tested microbial is determined by reading the first well in each drug series that contains no visible microbial growth, represented by well turbidity. The susceptibility of the microbial to each drug is determined based on the MIC as per the above color-coded table.

Abbreviation	Drug Name
AM	Ampicillin
A/S	Ampicillin-Sulbactam
TMP/SMX	Trimethoprim-sulfamethoxazole
CPM	Cefepime
MPM	Meropenem
GM	Gentamicin
ETP	Ertapenem
TB	Tobramycin
AK	Amikacin
CZ	Cefazolin
CTRX	Ceftriaxone
CAZ/AVI	Ceftazidime-Avibactam
CAZ	Ceftazidime
PIP/TAZ	Pipercillin-tazobactam
IPM	Imipenem
CIP	Ciprofloxacin
LVX	Levofloxacin

Supplementary Table S2. List of drugs and drug combinations prepared for the Gram-negative microtiter plate along with the common abbreviations used in our mobile application.
